# Metacognition in Auditory Distraction: How Expectations about Distractibility Influence the Irrelevant Sound Effect

**DOI:** 10.5334/joc.3

**Published:** 2017-11-30

**Authors:** Jan Philipp Röer, Jan Rummel, Raoul Bell, Axel Buchner

**Affiliations:** 1 Witten/Herdecke University, DE; 2 Heidelberg University, DE; 3 Heinrich Heine University Düsseldorf, DE

**Keywords:** metacognition, cognitive control, working memory, short-term memory, music cognition, emotion and cognition

## Abstract

Task-irrelevant, to-be-ignored sound disrupts serial short-term memory for visually presented items compared to a quiet control condition. We tested whether disruption by changing state irrelevant sound is modulated by expectations about the degree to which distractors would disrupt serial recall performance. The participants’ expectations were manipulated by providing the (bogus) information that the irrelevant sound would be either easy or difficult to ignore. In Experiment 1, piano melodies were used as auditory distractors. Participants who expected the degree of disruption to be low made more errors in serial recall than participants who expected the degree of disruption to be high, independent of whether distractors were present or not. Although expectation had no effect on the magnitude of disruption, participants in the easy-to-ignore group reported after the experiment that they were less disrupted by the irrelevant sound than participants in the difficult-to-ignore group. In Experiment 2, spoken texts were used as auditory distractors. Expectations about the degree of disruption did not affect serial recall performance. Moreover, the subjective and objective distraction by irrelevant speech was similar in the easy-to-ignore group and in the difficult-to-ignore group. Thus, while metacognitive beliefs about whether the auditory distractors would be easy or difficult to ignore can have an effect on task engagement and subjective distractibility ratings, they do not seem to have an effect on the actual degree to which the auditory distractors disrupt serial recall performance.

Irrelevant auditory information is ubiquitous in our everyday lives. This is not merely inconvenient, since it may also have a marked disruptive effect on cognitive performance (Beaman, Hanczakowski & Jones, 2014; Elliott et al., 2016; Hughes & Marsh, 2017; Ljungberg, Parmentier, Jones, Marsja & Neely, 2014; Marsh, Röer, Bell & Buchner, 2014; Vachon, Labonté & Marsh, 2017). However, not every background sound is likely to be recognized as a potential source of disruption. To illustrate, if one freely chooses to listen to music while performing a task, the degree of disruption that this sound is expected to have may be much lower than if it is the neighbors’ music permeating the walls that cannot be controlled. Do these expectations about a sound’s disruptive potential affect the degree to which such a sound actually disrupts performance? The extent to which metacognitive processes influence the subjective and objective distraction by irrelevant sound remains largely unexplored. In the present study, we address this question by experimentally manipulating participants’ expectations about the auditory distractors’ disruptive potential.

Short-term serial recall is the standard paradigm to measure the disruptive effect of auditory distractors on cognitive performance. In a typical experiment, participants must recall lists of visually presented items (usually digits) in the order of their presentation. When task-irrelevant, to-be-ignored background sound is played, serial recall performance is reduced compared to a quiet control condition (Colle & Welsh, 1976; see also Elliott, 2002; Ljungberg, Parmentier, Hughes, Macken & Jones, 2012; Marsh, Hughes & Jones, 2009; Röer, Bell & Buchner, 2013; Salamé & Baddeley, 1982). This reduction is largely independent of the auditory distractors’ sound level (Ellermeier & Hellbrück, 1998), and of whether the distractors are played during item encoding or maintenance (Jones, Madden & Miles, 1992; Röer, Bell & Buchner, 2014c). Distractor sequences that contain many abrupt changes in the amplitude or frequency spectrum such as music or speech have been found to have a particularly large disruptive effect on serial recall performance (Hughes, Tremblay & Jones, 2005; Jones et al., 1992; Röer, Bell, Marsh & Buchner, 2015; Schlittmeier, Weißgerber, Kerber, Fastl & Hellbrück, 2012). In some studies, a sequence of repeated sounds is used instead of a quiet control condition. The increase in disruption by changing sounds relative to repeated sounds can be referred to as the changing state irrelevant sound effect (cf. Hughes et al., 2005; Jones et al., 1992).

Thus far, metacognitive beliefs about the degree of disruption have not been manipulated directly. However, several studies have examined how top-down control processes and expectations influence the irrelevant sound effect (e.g., Alley & Greene, 2008; Hughes, Hurlstone, Marsh, Vachon & Jones, 2013; Vachon, Hughes & Jones, 2012). Perham and Vizard (2011), for instance, investigated whether the preference for background music mediates the disruptive effect of irrelevant sound. To this end, they compared the disruption of serial recall by music that the participants liked with that of music that they disliked. A typical irrelevant sound effect was found in the form of a significant reduction of serial recall performance relative to the quiet control condition. This effect, however, was independent of preference. After the experiment, participants were asked to rate the perceived distractibility. Interestingly, participants reported to have been similarly disrupted by the liked and the disliked music.

Furthermore, individual differences in working memory capacity (WMC) do not predict the magnitude of the irrelevant sound effect. This is relevant, because WMC is thought to reflect the ability to constrain attention to the focal task in the presence of irrelevant information. If top-down control processes that facilitate task engagement (as measured by WMC) exert influence on disruption, individuals with low WMC should be more distracted by irrelevant sound than individuals with high WMC. In several studies, however, individual differences in WMC were unrelated to the susceptibility to auditory distraction by changing state irrelevant sound (Beaman, 2004; Elliott & Briganti, 2012; Hughes et al., 2013; Körner, Röer, Buchner & Bell, 2017; Röer, Bell, Marsh, et al., 2015; Röer, Körner, Buchner & Bell, 2017; Sörqvist, 2010; Sörqvist, Marsh & Nöstl, 2013).

By looking at whether the disruption of serial recall performance decreases after preexposure to the irrelevant sound or over the course of the experiment, it can be measured to what extent participants habituated to the distractor sequences. The literature on habituation to irrelevant sound, however, is rather inconsistent. Some studies have found evidence of habituation (Bell, Röer, Dentale & Buchner, 2012; Pelletier, Hodgetts, Lafleur, Vincent & Tremblay, 2016; Röer, Bell & Buchner, 2014a, 2014b), others have not (Ellermeier & Zimmer, 1997; Jones, Macken & Mosdell, 1997; Röer, Bell, Dentale & Buchner, 2011). While these findings do suggest that expectations based on the preceding auditory stimulation can have a beneficial effect on performance, this is apparently not always the case. Amongst other factors, the likelihood of finding habituation effects seems to depend on the complexity of the distractor material and the choice of the experimental design (for an overview, see Röer et. al., 2014).

Hughes et al. (2013) examined whether disruption by changing state irrelevant sound is sensitive to top-down influences by manipulating the difficulty with which the visually presented items could be identified. In the easy-to-encode condition, the to-be-remembered items were clearly visible, because they were presented in black font on a white background. In the difficult-to-encode condition, the perceptual discriminability of the to-be-remembered items was reduced by adding static Gaussian noise. This visual degradation of the relevant information, however, had no effect on the changing state irrelevant sound effect. In both conditions, serial recall was similarly disrupted relative to a sequence of repeated sounds. In another experiment of the same study, the authors tested whether the magnitude of disruption would be modulated by foreknowledge of the upcoming distractor sequence. To this end, a visual notice informed participants on some trials whether the upcoming distractor sequence would be a changing sound or a repeated sound. This stimulus-unspecific warning had no effect on serial recall performance. By contrast, when auditory deviant sounds were used instead of changing state sounds (Hughes et al., 2013), or when stimulus-specific foreknowledge was provided in form of a visual transcript of the upcoming distractor sequence (Bell, Röer, Marsh, Storch & Buchner, in press; Röer, Bell & Buchner, 2015), disruption was significantly decreased.

In none of the studies summarized above, however, the expected degree of disruption was manipulated directly. Although the evidence thus far seems to suggest that unspecific expectations about the upcoming distraction do not influence the irrelevant sound effect (Hughes et al., 2013; Röer, Bell & Buchner, 2015), it is also possible that previous manipulations were too subtle to modulate disruption. For instance, the impact of unspecific foreknowledge depends on the effectiveness with which participants are able to adjust their task engagement based on the perceived degree of disruption in previous trials. However, it has been repeatedly found that subjective distractibility ratings predict only a small portion of the disruption actually observed (Beaman, 2005; Ellermeier & Zimmer, 1997). Thus, the effect of an adjustment of task engagement based on the subjective distractibility is limited from the outset. It also remains unclear whether previous findings can be generalized to a situation in which the participants do not expect the auditory distractors to disrupt performance at all.

Given this, our aim in the present study was to provide a direct test of the influence of metacognitive processes on the subjective and objective distraction by irrelevant sound. In Experiment 1, piano melodies were used as auditory distractors. We expected that participants’ metacognitive beliefs about the effects of music can be manipulated most effectively, because many people listen to music by choice when performing a task (cf. Perham & Vizard, 2011). Participants were instructed either that previous research suggested that the music would not distract them at all from their task or that the music will severely distract them from their task. This procedure was adapted from a recent study in which expectations about task demands significantly affected the allocation of attentional resources between two simultaneously to-be-maintained goals (Rummel & Meiser, 2013). Furthermore, we asked the participants to rate the perceived level of difficulty after the experiment in order to explore the degree to which expectations had influenced subjective distractibility.

## Ethics Statement

The study was approved by the ethics committee of the Faculty of Mathematics and Natural Sciences at Heinrich Heine University Düsseldorf. Written informed consent was obtained before the experiment.

## Experiment 1

### Method

#### Participants

A total of 106 students at Heinrich Heine University (72 women) were paid or received course credit for participating in the experiment (*n* = 53 in the easy-to-ignore and in the difficult-to-ignore group). Their ages ranged from 18 to 48 years (*M* of age = 23). All participants reported normal hearing and normal or corrected-to-normal vision.

#### Materials

A standard serial recall task was used. For each trial, eight to-be-remembered digits were sampled randomly without replacement from the set {1, 2, …, 9}. Digits were presented at a rate of 1 Hz (800 ms on, 200 ms off) in black font on a white background in the centre of the computer screen. From a viewing distance of 50 cm, digits subtended a vertical visual angle of 1.34° and a horizontal angle of 0.83°.

Auditory distractors were eight piano melodies in the key of C major that were taken from Röer, Bell, and Buchner (2014a). The melodies were unfamiliar to the participants. Distractor sequences lasted 8 s, and were normalized to minimize amplitude differences. Auditory distractors were played binaurally at 65 dB(A) using headphones with high-insulation hearing protection covers.

#### Procedure

Participants were randomly assigned to one of two expectation conditions (easy-to-ignore, difficult-to-ignore) that differed only in the written instructions that appeared on the computer screen before the experiment proper began. The descriptions for the conditions were the German translations of the following instructions:

*Easy-to-ignore*. During some trials, music will be presented over headphones. This music is completely irrelevant and should be ignored. Previous research suggests that the music will not distract you from your task. Many participants found it easier to concentrate on the task when music was played.

*Difficult-to-ignore*. During some trials, music will be presented over headphones. This music is completely irrelevant and should be ignored. Previous research suggests that the music will severely distract you from your task. Many participants found it more difficult to concentrate on the task when music was played.

In all other aspects, the experiment was the same for both groups. Participants completed a total number of 24 trials which were divided into two blocks. The training block consisted of eight quiet trials. The experimental block consisted of eight quiet trials and eight distractor trials, in which irrelevant music was played during the presentation of the to-be-remembered digits. In each trial, a different distractor sequence was presented. Immediately after the presentation of the to-be-remembered digits, eight question marks appeared on the screen. Participants used the number pad of the keyboard to replace the question marks with the to-be-remembered digits in the order in which they had been presented. It was possible to skip over a serial position by pressing a “don’t know” button on the keyboard, but it was not possible to correct a response. After completion of the serial recall task, participants were asked to indicate the perceived level of difficulty in the quiet condition and in the irrelevant music condition on a six-point scale, with 1 indicating “very easy” and 6 indicating “very difficult”. The experiment took approximately 20 minutes.

#### Design

A mixed design was used, with expectation (easy-to-ignore, difficult-to-ignore) as the between-subjects variable and auditory condition (quiet, irrelevant music) as the within-subjects variable. The dependent variable was serial recall performance. Answers were scored according to a strict serial recall criterion. A response was only scored as correct when the digit was reproduced in the exact serial position in which it had been presented. For within-subject comparisons, a multivariate approach was used. In the present application, all multivariate test criteria correspond to the same exact *F* statistic, which is reported. The level of α was set to .05 for all analyses. Partial Eta-squared (denoted as η_p_^2^) is reported as a measure of effect size.

#### Power analysis

Of primary interest was the question of whether there is an interaction of the between-subjects variable and the within-subject variable. If expectations influence the irrelevant sound effect through metacognitive processes, the difference in recall performance between the quiet and the irrelevant music condition should differ as a function of the expected degree of disruption. Given a total sample size of *N* = 106, and α = β = .05, interaction effects of size *f*(V) = 0.47 could be detected. All power calculations reported in this article were conducted using G*Power (Faul, Erdfelder, Lang & Buchner, 2007).

### Results

**Figure 1:**
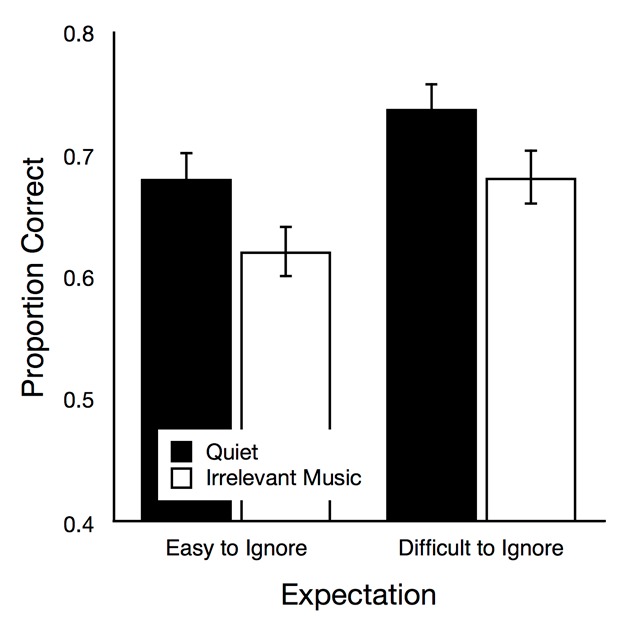
Recall performance as a function of auditory condition (quiet, irrelevant music) in the easy-to-ignore group and in the difficult-to-ignore group. The error bars represent the standard errors of the means.

*Serial recall*. Figure 1 shows serial recall performance as a function of expectation and auditory condition. There were main effects of group, *F*(1,104) = 4.25, *p* = .042, η_p_^2^ = .04, and of auditory condition, *F*(1,104) = 25.70, *p* < .001, η_p_^2^ = .20. The interaction of these variables was not significant, *F*(1,104) = 0.04, *p* = .845, η_p_^2^ < .01. When the data of both groups were analyzed separately, a typical irrelevant sound effect (i.e., reduced recall performance in the irrelevant music condition relative to the quiet condition) was found in both the easy-to-ignore group, *t*(52) = 3.64, *p* = .001, η_p_^2^ = .20, and in the difficult-to-ignore group, *t*(52) = 3.52, *p* = .001, η_p_^2^ = .19.

**Figure 2:**
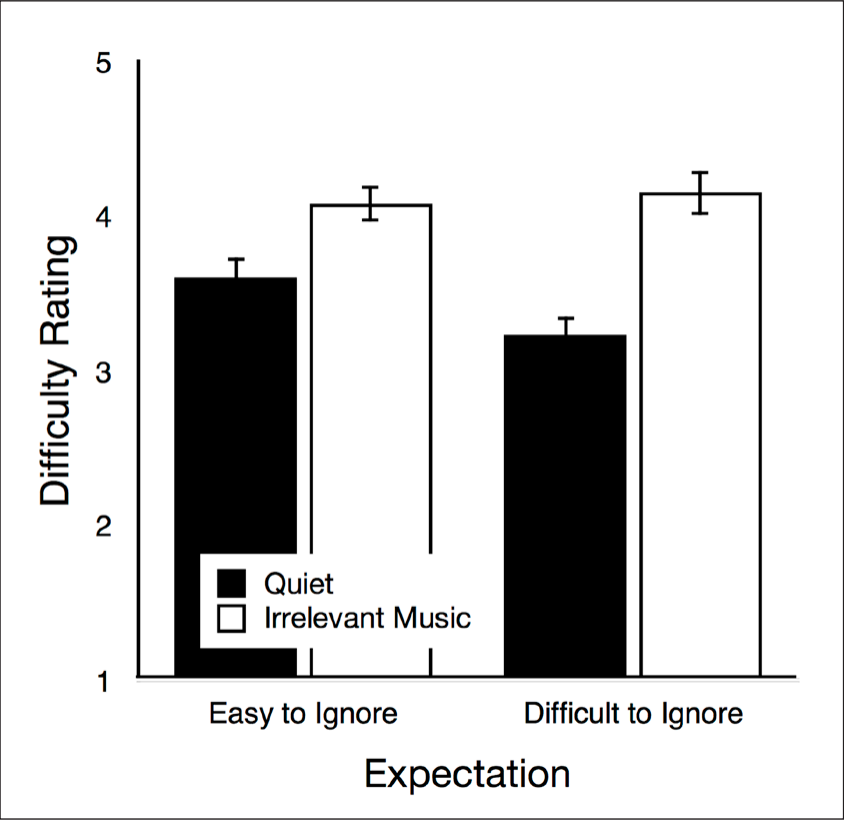
Difficulty rating as a function of auditory condition (quiet, irrelevant music) in the easy-to-ignore group and in the difficult-to-ignore group. The error bars represent the standard errors of the means.

*Difficulty rating*. In Figure 2, subjective difficulty ratings illustrate how difficult the participants perceived the task to be in the quiet condition and in the irrelevant music condition. The main effect of group was not significant, *F*(1,104) = 0.92, *p* = .339, η_p_^2^ = .01, but there was a significant main effect of auditory condition, *F*(1,104) = 43.01, *p* < .001, η_p_^2^ = .29. Interestingly, there was also a significant interaction of group and auditory condition, *F*(1,104) = 4.52, *p* = .036, η_p_^2^ = .04, reflected by the fact that the difference in difficulty between the quiet condition and in the irrelevant music condition was perceived to be higher in the difficult-to-ignore group than in the easy-to-ignore group. When the data of both groups were analyzed separately, trials with irrelevant music were rated to be significantly more difficult than quiet trials in both groups, but the effect was less pronounced in the easy-to-ignore group, *t*(52) = 3.27, *p* = .002, η_p_^2^ = .17, compared to the difficult-to-ignore group, *t*(52) = 5.90, *p* < .001, η_p_^2^ = .40.

### Discussion

In Experiment 1, expectations about the degree of disruption had no impact on the irrelevant sound effect on serial recall. The magnitude of disruption was similar regardless of whether participants expected the auditory distractors to be easy or difficult to ignore. However, participants who expected the degree of disruption to be low made more errors in recall than participants who expected the degree of disruption to be high, suggesting that participants implicitly or explicitly adjusted their task engagement to the expected level of difficulty. When asked after the experiment to rate the difficulty of the conditions, participants in the easy-to-ignore group rated the disruptive effect of irrelevant sound (i.e., the difference in difficulty between the quiet condition and the irrelevant music condition) to be smaller than participants in the difficult-to-ignore group. These results show that the manipulation of metacognitive processes was successful: The overall performance difference between groups indicates that participants adjusted their task engagement to compensate for the expected difficulty. Specifically, participants in the easy-to-ignore group expected the task to be easier than participants in the difficult-to-ignore group. What is more, this expectation also had an effect on the subjective difficulty ratings.

The finding that the actual magnitude of disruption on serial recall performance was unaffected by expectations is consistent with previous studies, in which top-down control processes did not influence the changing state irrelevant sound effect. For instance, an unspecific warning about whether in the upcoming trial a changing sound (i.e., a distractor sequence that is difficult to ignore) or a repeated sound (i.e., a distractor sequence that is easy to ignore) would be presented had no beneficial effect on performance relative to a condition without such a warning (Hughes et al., 2013; Röer, Bell & Buchner, 2015). The present findings, however, extend that of previous studies in two ways. First, it was demonstrated that expectations can have a metacognitively mediated effect on performance in that participants in the difficult-to-ignore group made fewer errors overall than participants in the easy-to-ignore group, suggesting that participants who expected the task to be easy generally engaged in task performance to a lesser degree than participants who expected the task to be difficult. Second, this difference in expectation was also reflected in the subjective difficulty ratings. Participants who expected the degree of disruption to be low reported a smaller subjective difference in difficulty between the quiet and the irrelevant music condition than participants who expected the degree of disruption to be high.

In Experiment 2, we examined whether metacognitive processes also have an effect on task engagement and subjective distractibility when the irrelevant music is replaced by irrelevant speech. In Experiment 1, piano melodies were used as auditory distractors, because many people listen to music by choice when performing a task (Perham & Vizard, 2011). Indeed, listening to music may benefit sport performance and other largely automatic tasks (for a recent meta-analysis of the impact of background music on a variety of tasks, see Kämpfe, Sedlmeier & Renkewitz, 2010), and it has a positive effect on mood and arousal. Thus, many people are probably not fully aware of the detrimental effect of background music on memory. Irrelevant speech, by contrast, is typically perceived as highly disruptive (e.g., Ellermeier & Zimmer, 1997). This is not surprising, because natural speech (Ellermeier, Kattner, Ueda, Duomoto & Nakajima, 2015; Wöstmann & Obleser, 2016) and sequences that are generated to resemble natural speech in its acoustic parameters (Tremblay, Nicholls, Alford & Jones, 2000) are among the most potent auditory distractors that are currently known. Using irrelevant speech thus allowed us to explore possible boundary conditions of the effect of expectations observed in Experiment 1.

## Experiment 2

### Method

#### Participants

A total of 74 students at Heinrich Heine University (41 women) were paid or received course credit for participating in the experiment (*n* = 37 in the easy-to-ignore and in the difficult-to-ignore group). Their ages ranged from 18 to 39 years (*M* of age = 23). All participants reported normal hearing and normal or corrected-to-normal vision.

#### Materials, Procedure, and Design

Materials, Procedure, and Design were identical to Experiment 1, with the following exceptions. Auditory distractors were eight texts spoken by a male speaker that were taken from Bell et al. (2012). Distractor sequences lasted 8 s, and were normalized to minimize amplitude differences. The descriptions for the conditions were the German translations of the following instructions.

*Easy-to-ignore*. During some trials, speech will be presented over headphones. This speech is completely irrelevant and should be ignored. Previous research suggests that the speech will not distract you from your task. Many participants found it easier to concentrate on the task when speech was played.

*Difficult-to-ignore*. During some trials, speech will be presented over headphones. This speech is completely irrelevant and should be ignored. Previous research suggests that the speech will severely distract you from your task. Many participants found it more difficult to concentrate on the task when speech was played.

In each distractor trial, a different spoken text was presented. The experiment took approximately 20 minutes.

#### Power analysis

Given a total sample size of *N* = 74, and α = β = .05, interaction effects of size *f*(V) = 0.57 could be detected.

### Results

**Figure 3:**
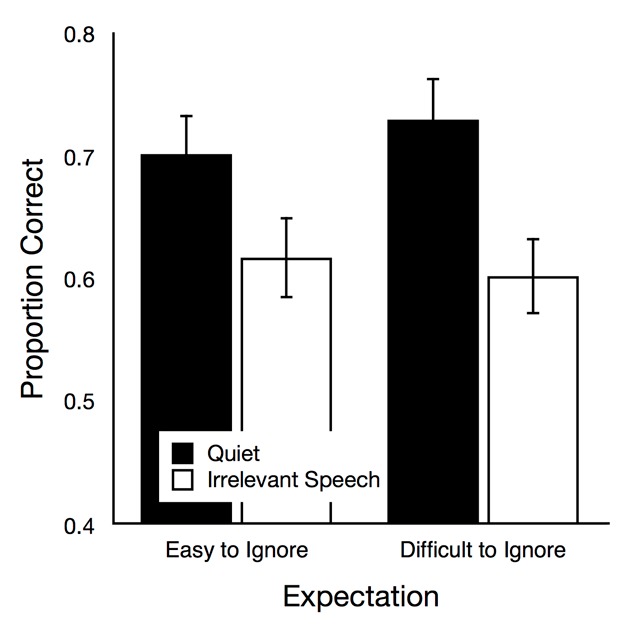
Recall performance as a function of auditory condition (quiet, irrelevant speech) in the easy-to-ignore group and in the difficult-to-ignore group. The error bars represent the standard errors of the means.

*Serial recall*. In Figure 3, serial recall performance is illustrated as a function of auditory condition and group. The main effect of group was not significant, *F*(1,72) = 0.03, *p* = .874, η_p_^2^ < .01, but there was a significant main effect of auditory condition, *F*(1,72) = 53.35, *p* < .001, η_p_^2^ = .43. The interaction of these variables was not significant, *F*(1,72) = 2.25, *p* = .138, η_p_^2^ = .03. When the data of both groups were analyzed separately, a typical irrelevant speech effect (i.e., reduced recall performance in the irrelevant speech condition relative to the quiet condition) was found in both the easy-to-ignore group, *t*(36) = 4.28, *p* < .001, η_p_^2^ = .34, and in the difficult-to-ignore group, *t*(36) = 5.99, *p* < .001, η_p_^2^ = .50.

**Figure 4:**
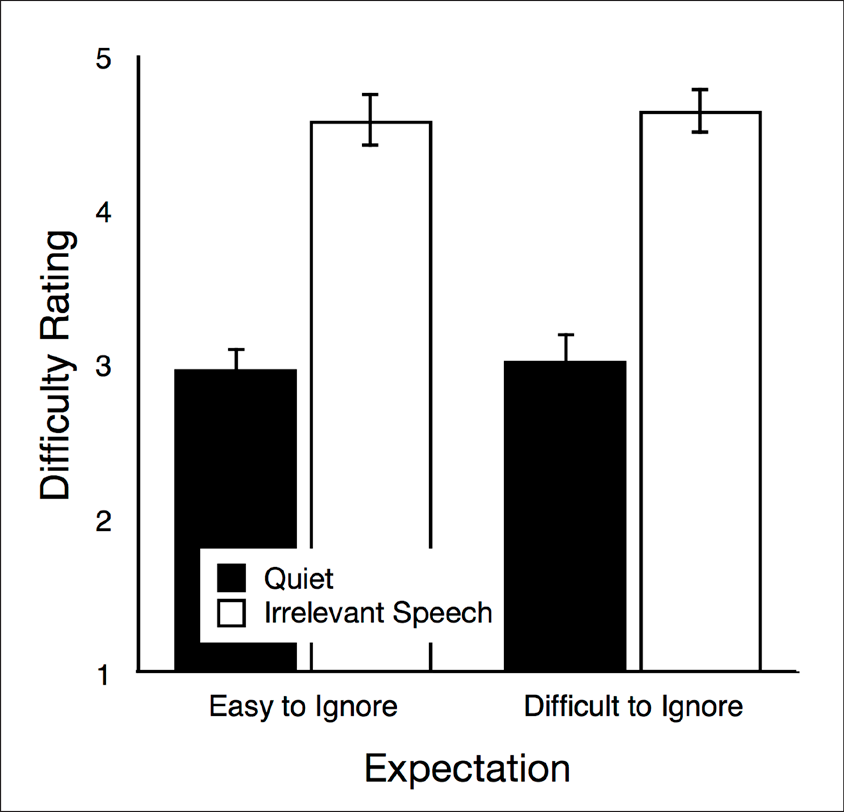
Difficulty rating as a function of auditory condition (quiet, irrelevant speech) in the easy-to-ignore group and in the difficult-to-ignore group. The error bars represent the standard errors of the means.

*Difficulty rating*. Figure 4 illustrates how difficult the participants perceived the task to be, depending on group and auditory condition. The main effect of group was not significant, *F*(1,72) = 0.08, *p* = .782, η_p_^2^ < .01, but there was a significant main effect of auditory condition, *F*(1,72) = 201.87, *p* < .001, η_p_^2^ = .74. The interaction of group and auditory condition was not significant, *F*(1,104) = 0.00, *p* = 1, η_p_^2^ = .00 (in fact, the difference between the perceived difficulty in the quiet and irrelevant speech condition was numerically identical in both groups). When the data of both groups were analyzed separately, trials with irrelevant speech were rated to be significantly more difficult than quiet trials in both the easy-to-ignore group, *t*(36) = 11.91, *p* < .001, η_p_^2^ = .80, and in the difficult-to-ignore group, *t*(36) = 8.85, *p* < .001, η_p_^2^ = .69.

### Discussion

Experiment 2 replicates the main finding of Experiment 1 that expectations about the degree of disruption had no impact on the irrelevant sound effect on serial recall. Whether participants were instructed that the irrelevant speech would be easy or difficult to ignore did not affect the actual disruption that the auditory distractors produced. Unlike in Experiment 1, there was no main effect of group on serial recall performance. Thus, there was no evidence that participants adjusted their task engagement in response to the expected level of difficulty. Moreover, difficulty ratings were independent of whether participants expected the degree of disruption to be high or low, suggesting that participants’ beliefs about the distracting effect of irrelevant speech on cognitive performance are more accurate, and less susceptible to, (bogus) information about the to-be-expected difficulty than their beliefs about the distracting effect of irrelevant music.

## General Discussion

The present study provides evidence that expectations about the disruption of serial recall by irrelevant sound can have an effect on subjective and objective task performance. In Experiment 1, participants who expected the degree of disruption to be high performed better overall than participants who expected the degree of disruption to be low. Interestingly, the difference in subjective distractibility between the quiet and the irrelevant sound condition was higher in the difficult-to-ignore group than in the easy-to-ignore group. This effect, however, was only observed with background music. In Experiment 2, when background speech was used as to-be-ignored material, overall performance and subjective distractibility ratings were unaffected by the to-be-expected difficulty, suggesting that participants were aware that speech is highly disruptive and could not be convinced otherwise. In both experiments, the irrelevant sound effect on serial recall was independent of whether participants were instructed that the auditory distractors would be easy or difficult to ignore.

The latter finding is consistent with previous studies that examined the influence of top-down control processes and expectations on the magnitude of disruption by irrelevant sound compared to a steady state or quiet control condition. For instance, it has been repeatedly found that individual differences in the ability to constrain attention to the focal task are unrelated to the susceptibility to the changing state irrelevant sound effect (Beaman, 2005; Elliott & Briganti, 2012; Körner et al., 2017; Röer, Bell, Marsh, et al., 2015; Sörqvist, 2010). Disruption was also independent of the difficulty with which the visually presented target items could be encoded (Hughes et al., 2013) and of whether or not a visual notice informed participants whether the upcoming distractor sequence would be a changing sound or a repeated sound (Hughes et al., 2013; Röer, Bell & Buchner, 2015). Notable exceptions are studies in which participants were given specific advance information about the distractor sequence. The irrelevant sound effect, for instance, was significantly reduced when participants were given the opportunity to listen to the upcoming distractor sequence before the trial during a preexposure phase (Bell et al., 2012). A reduction was also found when a visual transcript of the upcoming distractor sequence was provided compared to a condition without foreknowledge (Bell et al., in press; Röer, Bell & Buchner, 2015). However, a foreknowledge effect was only observed with meaningful, syntactically coherent speech, and not with a randomly assembled list of words. This suggests that performance in the presence of auditory distraction may benefit from expectations, but for this beneficial effect to occur the advance information must be stimulus-specific and translatable into a predictive representation of the upcoming distraction. While unspecific expectations such as whether the distractors will be easy or difficult to ignore may have an effect on global task engagement—as was the case in Experiment 1—they are insufficient to reduce the unpredictability of the upcoming distraction, because they do not help to establish a stable internal representation of the distractor sequence (cf. Cowan, 1995).

Whether or not expectations about the degree of disruption metacognitively modulate the irrelevant sound effect is also of theoretical interest. According to the duplex-mechanism account (Hughes, 2014), there are two distinct forms of auditory distraction, which differ in their amenability to top-down control. While changing state sounds disrupt serial recall because the processing of the irrelevant order information in the distractor sequence interferes with the processing of the relevant order information in the item sequence, auditory deviant sounds disrupt serial recall because they capture attention and draw processing resources away from the focal task. The auditory deviant effect is assumed to be under top-down control. The changing state effect, however, occurs automatically and cannot be controlled. Thus, the duplex-mechanism account predicts that the disruptive effect of changing state irrelevant sound should be independent of whether participants expect it to be low or high. This was confirmed by the present results. Whether the auditory deviant effect is sensitive to participants’ metacognitive beliefs, or not, remains an open empirical question. It could certainly be argued that the duplex-mechanism account predicts that the disruptive effect of auditory deviant sounds should be affected by whether participants expect it to be low or high, but it is unclear whether such a phenomenon can be demonstrated empirically. Whereas Experiment 1 shows that it is possible to manipulate the participants’ beliefs about the distracting effect of music, it might be much more difficult to convince participants that an auditory deviant sound will not be distracting at all (and even more so that it will have a beneficial effect on task performance). Thus, it is doubtful whether such a manipulation can be effective.

Another interesting aspect of the present study is that participants who expected the degree of disruption to be high also perceived the difference between the quiet and the irrelevant sound condition to be more disruptive than participants who expected the degree of disruption to be low—at least when piano melodies were used as auditory distractors. This finding once again indicates that subjective distractibility ratings—just like subjective judgments of other mental states such as fatigue (cf. Schmidt et al., 2009)—have to be interpreted with caution as they do not necessarily speak to the disruption actually observed (Beaman, 2005; Ellermeier & Zimmer, 1997). By implication, this may also suggest that people are not always aware of the fact that background music is harmful to cognitive performance. This may well be different for the detrimental effects of background speech, probably because listening to music has a positive effect on mood and arousal (cf. Schellenberg, 2012) which background speech generally lacks. The finding that the mere expectation about whether a sound will be easy or difficult to ignore has a direct effect on perceived distractibility is also of practical relevance (e.g., at the workplace, in the classroom). While it seems that top-down control processes are unable to reduce the disruptive effect of changing background sound, expectations about the degree of disruption may lead to an increase in task engagement and with that benefit performance after all. Thus, it is important to educate, for example, open-plan office workers and schoolchildren about the harmful effect of irrelevant sound to help them build up realistic expectations about the degree of disruption and adjust task engagement accordingly or, even better, take precautions to protect themselves from irrelevant sound.
